# Production and characterization of novel marine black yeast’s exopolysaccharide with potential antiradical and anticancer prospects

**DOI:** 10.1186/s12934-024-02332-1

**Published:** 2024-02-22

**Authors:** Eman H. Zaghloul, Hala H. Abdel-Latif, Asmaa Elsayis, Sahar W.M. Hassan

**Affiliations:** National Institute of Oceanography and Fisheries (NIOF), Cairo, Egypt

**Keywords:** Black yeast, Exopolysaccharide, *Hortaea Werneckii*, Anticancer, Chemical characterization

## Abstract

The marine black yeasts are characterized by the production of many novel protective substances. These compounds increase their physiological adaptation to multi-extreme environmental stress. Hence, the exopolysaccharide (EPS) producing marine black yeast SAHE was isolated in this study. It was molecularly identified as *Hortaea werneckii* (identity 98.5%) through ITS1 and ITS4 gene sequencing analysis. The physicochemical properties of the novel SAHE-EPS were investigated through FTIR, GC-MS, TGA, ESM, and EDX analysis, revealing its heteropolysaccharide nature. SAHE-EPS was found to be thermostable and mainly consists of sucrose, maltose, cellobiose, lactose, and galactose. Furthermore, it exhibited an amorphous texture and irregular porous surface structure. SAHE-EPS showed significant antiradical activity, as demonstrated by the DPPH radical scavenging assay, and the IC50 was recorded to be 984.9 μg/mL. In addition, SAHE-EPS exhibited outstanding anticancer activity toward the A549 human lung cancer cell line (IC50 = 22.9 μg/mL). Conversely, it demonstrates minimal cytotoxicity toward the WI-38 normal lung cell line (IC50 = 203 μg/mL), which implies its safety. This study represents the initial attempt to isolate and characterize the chemical properties of an EPS produced by the marine black yeast *H. werneckii* as a promising antiradical and anticancer agent.

**Supplementary Information:**

The online version contains supplementary material available at 10.1186/s12934-024-02332-1.

## Introduction

Polysaccharides are valuable biopolymers retrieved from various natural origins, including bacteria, fungi, seaweeds, plants, and more [1–3]. The high molecular weight carbohydrate polymers that are released extracellularly by microorganisms to the environment, known as microbial exopolysaccharides (EPS), are believed to be harmonized through chemical reactions that involve intracellular sugars condensation occurring during the growth and metabolic processes of the microorganisms [1, 4, 5]. Among the natural sources of polysaccharides, microbial ones are superior to other sources in several ways, including outstanding structural consistency. Additionally, the probability of accomplishing a high EPS output by applying synthetic biology techniques and improving culture conditions is a promising and emerging research field [6, 7].

Microorganisms within numerous taxonomic groups can produce EPS with remarkable physicochemical and functional characteristics [8]. However, yeast-produced EPS exhibit various preferences such as low expense, non-toxicity, and being eco-friendly. In addition, they are appealing for large-scale manufacturing since they can be separated from the growth media more readily than those produced by bacterial cells [8, 9] EPS have been reported to be made up to this point by yeasts belonging to different genera of *Hansenula*, *Aureobasidium*, *Bullera*, *Debaryomyces*, *Lipomyces*, *Pichia*, *Candida*, *Trichosporon*, *Pseudozyma*, *Rhodotorula*, *Kluyveromyces*, *Hanseniaspora*, *Metschnikowia*, *Sporobolomyces*, *Phomopsis*, *Exophiala*, *Tremella*, *Kazachstania*, *Clavispora*, and *Cryptococcus* [10–13].

Since EPS polymers are a significant element affecting survival in extreme environments, marine microorganisms’ ability to synthesize peculiar EPS with various biological activities is enhanced by their exceptional living conditions, including high pressure, low temperature, high salinity, and oligotrophic conditions [14, 15]. The structural and compositional variations, as well as the biological activities exhibited by marine EPS, hold potential for advantageous applications in the field of medicinal and pharmaceutical industries [16, 17]. Moreover, the multifunctional properties of these substances, including their ability to stabilize, thicken, gel, and emulsify, make them highly valuable in various sectors, including but not limited to the food, cosmetics, pharmaceutical, and petroleum industries [6, 18]. For example, the fungal EPS Scleroglucan is used in industrial applications to enhance oil recovery. Scleroglucan, also known as Schizophyllan, is used under the trade name BIOVIS, developed by Degussa society, in the manufacturing of drilling fluids [19]. Farwick et al. [20] documented the significant water retention ability of Scleroglucan on epithelial cells. Additional industrial uses for this EPS included the formulation of adhesives, watercolors, printing inks, and animal feed composition. Moreover, it finds use in the production of cosmetics as well as in a range of skincare products, including creams and protective lotions [20].

Despite all mentioned, due to their reduced yield and elevated production costs, these polysaccharides constitute a limited portion of the present polymer’s market [6]. Due to advancements in analysis techniques and separation approaches, marine EPS from microorganisms have become a new hub for investigation [21, 22]. Since these extracellular polymers are one of the critical factors that can aid in overcoming harsh environmental circumstances, it is intriguing to inquire about the ability to produce such significant biomolecules with various applications in industrial and medical applications, such as anticancer treatments with bifidogenic properties, plasma expanders, and pharmaceutical excipients. Moreover, EPS is used in many surfactant, food, and packaging edible film industries [5].

Among the marine fungi is an exclusive group of black yeasts that can yield vital metabolites essential in overcoming their surroundings’ harsh conditions. Black yeasts are polymorphic, polyextremotolerant fungi that have filamentous and meristematically like yeasts mode of growth and can thrive in vast adverse environmental conditions, such as high temperature, limited nutrient accessibility, osmotic or mechanical stress, as well as acidic, alkaline, and toxic environments [23, 24].

The marine black yeast “*Hortaea werneckii”* is an extremely halotolerant species that can flourish through a broad spectrum of NaCl [25]. *H. werneckii* is classified within the Capnodiales order of the Ascomycota phylum [26]. It has been previously isolated from marine samples. Elsayed et al. [26] reported *H. werneckii* isolation from salt marsh, and Brauers et al. [27] reported *H. werneckii* isolation from Mediterranean sponge, while Plemenitaš et al. [28] reported its presence in hypersaline water in Europe. Additionally, it was found as a contaminant in Amazonian salted seafood, leading to food deterioration [29]. At the same time, *H. werneckii* isolation is not widely reported. This may be attributed to the fact that the process of isolation and purification of the black yeast *H. werneckii* is very challenging as it exists mainly in extreme habitats characterized by limited nutrients, detrimental UV radiation, low oxygen levels, osmotic stress, or a combination of these characteristics. It also grows slowly and needs a prolonged incubation period, which increases the chances of contamination with other fungi [30].

*H. werneckii* is a pathogenic yeast causing skin infections; it is the etiological agent causing tinea nigra [31], and it is not safe to be used directly for industrial applications. However, it is documented to produce several beneficial bioactive compounds, such as EPS and melanin, which are useful for UV protection, antioxidants, and wound healing. These derived bioactive compounds are safe and more suitable for cosmetics and pharmaceutical applications [32].

To the best of our knowledge, no existing documentation is available about the production of EPS by this particular yeast strain. Thus, the current study’s main intention was to produce, characterize, and investigate some bioactivity of the produced EPS from the locally isolated marine black yeast as an eco-friendly and economical source.

## Materials and methods

### Isolation of marine black yeast

Black yeast isolation was carried out from marine samples (water and sediments) collected from Alexandria International Coastal Road, Egypt (31.068709” N, 29.774321” E). Serial dilutions were prepared for each sample using sterile aged seawater. After that, one mL of each dilution was inoculated on Sabouraud dextrose agar (SDA) enriched with L-tyrosine, 150 mg/L chloramphenicol to suppress bacterial growth, and 10% NaCl [32]. Plates were incubated at 30 °C for 21 days. Black melanized colonies were picked up by a sterile platinum loop and were successively streaked on SDA for purification. Finally, pure isolates were examined under a light microscope and stored as glycerol stocks (20% v/v) at -20 °C for subsequent investigations.

### Molecular identification and phylogenetic analysis of isolate SAHE

Out of the obtained isolates, the marine isolate named SAHE showed a distinctive black mucoid appearance, indicating exopolysaccharide production [5]. It was examined under a scanning electron microscope (SEM) and molecularly identified through ITS1 and ITS4 gene sequencing analysis. Elim Biopharmaceuticals Company’s Pathogen spin DNA/RNA extraction kit (California, USA) was employed to extract the DNA from the experimental strain SAHE. ITS1 and ITS4 genes were amplified using the forward and reverse primer pair (5’- TCCGTAGGTGAACCTGCGG, − 3’) and (5’- TCC TCC GCT TAT TGA TAT GC, -3’), respectively. Then, the PCR reaction was conducted following the protocol (initial denaturation at 95 °C for 5 min; 30 cycles of denaturation at 94 °C for 40 s; annealing at 58 °C for 40 s, and extension at 72 °C for 40 s). In the end, the reaction was left for 5 min at 72 °C for final extension. The same primers were used to sequence the purified PCR product through Elim Biopharmaceuticals (USA) [33]. Basic Local Alignment Search Tool (BLAST), available through the National Center for Biotechnology Information’s (NCBI) website, was used to analyze the sequences and find DNA commonalities using Hall’s BioEdit sequence alignment editor (version 7.2.5). The Prodist-Neighbor-joining method (version 3.6a2.1) was employed for the phylogenetic analysis of ITS region sequences.

### EPS production

The new marine black yeast isolate SAHE, identified as *Hortaea werneckii* SAHE, was used for EPS production. Briefly, the SAHE seed culture was prepared by inoculation into a 250 mL Erlenmeyer flask containing 50 mL of the following medium (g/L):

sucrose, 30; (NH_4_)_2_SO_4_, 2.5; KH_2_PO_4_, 1; yeast extract, 1; MgSO_4_. 7 H_2_O, 0.5 and CaCl_2_ − 2H_2_O. 0.1 [34] was prepared using aged seawater at pH 5 and incubated at 30^o^C for 10 days in an orbital shaker (150 rpm). After incubation, 1% (v/v) of the prepared seed culture was used to inoculate 1 L of the same medium and incubated under the same conditions for EPS production.

### SAHE-EPS recovery

The yeast culture was centrifuged at (5,000 rpm) for 20 min to recover the cell-free supernatant. The gathered supernatant was mixed with 10% trichloro acetic acid (TCA) for 30 min to get rid of proteins, then centrifuged at (5,000 rpm) and pellets were disposed of. Two volumes of 95% chilled ethanol were mixed with the remaining supernatant and kept for 24 h at 4 °C to ensure precipitation of the EPS. Then, the precipitate was gathered by centrifugation at 5,000 rpm. The obtained pellets were dried at 30 °C overnight [35]. Deionized water was used to dissolve the precipitate, transmitted to dialysis bags (12–14 kDa) for 48 h, and freeze-dried to get the EPS. The final gained EPS after drying was weighed as 250 mg/L.

### Characterization and chemical analysis of SAHE-EPS

#### Morphological and elemental studies

About 10 mg of SAHE-EPS was examined under SEM (JSM-IT 200, Jeol, Japan) to determine its morphological properties and surface texture. Prior to visualization, the sample underwent a gold (15 Å) coating process using physical vapor deposition for 2 min. The visualization was performed at an accelerating voltage of 20.0 kV [36, 37]. The elemental analysis of SAHE-EPS was afterward conducted without any prior treatment utilizing a scanning electron microscope-energy dispersive X-ray (SEM-EDX) spectrometer. The X-rays that were emitted were used to ascertain the weight and atomic percentages of the recorded elements [38].

#### Fourier-transform infrared spectroscopy (FTIR)

FTIR spectroscopy analysis was conducted on the purified SAHE-EPS powder to detect its prominent and distinctive functional groups. The purified SAHE-EPS powder 10 mg was mixed with KBr, forming a pellet, and it was loaded onto the single crystal germanium of the FTIR spectrometer (Bruker, ALPHA, Germany). Following background subtraction, the spectral data was acquired within the wavenumber range of 4,000 to 400 cm^− 1^ at a spectral resolution of 4.0 cm^− 1^ [39, 40].

#### Gas chromatography-mass spectrometry (GC-MS)

The silylated glycosides that make up the sugar components of SAHE-EPS were identified by an acid hydrolysis process, a silylation derivatization step, and a GC-MS analysis [41, 42].

To perform acid hydrolysis, 20 mg of SAHE-EPS were treated with 3.0 mL of sulfuric acid (2 M) in a sealed glass tube. The mixture was heated to 105 °C for 10 h for complete hydrolysis [35]. Before being neutralized with barium carbonate (pH 7.0), the tube was cooled at room temperature. After that, the precipitate was eliminated by centrifugation, and the supernatant was filtered through a 20 μm syringe before being lyophilized. Then, the dried hydrolysates were silylated with 1:1 pyridine-BSTFA (N,O-bis(trimethylsilyl) trifluoroacetamide) (50 μL/mg) at 80 °C for 16 h. 2 μL of the derivatized sugars were injected into a GC-MS instrument (MassHunter GC-MS 1989–2014, Agilent Technologies, Inc.) using a previously established separation procedure. The temperatures of the detector and injector were consistently maintained at 320 °C. The column used was HP5MS, with dimensions of 30 m × 0.25 mm × 0.25 μm. Initially, the column was set at 100 °C for 1 min. It was then gradually increased from 100 to 260 °C at a rate of 4 °C for 1 min. Finally, it was held at 260 °C for 10 min. The carrier gas, helium, was adjusted to a 1 ml/min flow rate. The detected sugars were identified using the NIST library [36].

#### Thermal gravimetric analysis (TGA)

The polysaccharide was subjected to thermogravimetric analysis using a Perkin Elmer Diamond TGA system. A test material weighing 15 mg was used for the analysis. The TGA signal, expressed as a percentage change in weight, is plotted along the y-axis of the TGA curve, while the temperature of the reference material is represented on the x-axis. The EPS underwent a consistent rate of 20 °C per minute of heating within a platinum crucible. The procedure was executed under a nitrogen atmosphere and a temperature range of 26 to 500 °C. The resulting decrease in weight was measured and recorded [43].

### SAHE-EPS antiradical activity

The antiradical activity of SAHE-EPS was assessed using the 2,2-diphenyl-1-picryl-hydrazyl-hydrate (DPPH) assay. Briefly, SAHE-EPS was dissolved in DMSO at a concentration of 1000 μg/mL. Then, 100 μL of freshly prepared DPPH reagent was added to 100 μL of the sample or vitamin C. The plate was then shaken to ensure thorough mixing, wrapped in aluminum foil, and incubated at room temperature in the dark for 30 min. Subsequently, the color intensity was measured at a wavelength of 450 nm using an Optima spectrophotometer. The radical scavenging activity was calculated using the subsequent formula: DPPH inhibition (%) = (A_C_-A_E_)/A_C_) × 100, where A_C_ represents the absorbance of the control, and A_E_ represents the absorbance of the test. In addition, the IC50 value was determined using the formula Y = aX + C where Y = 50, a = slope, X = concentration of sample inhibit 50% of DPPH, and C = intercept [44, 45].

### SAHE-EPS superoxide anion scavenging activity

For confirmation of the antiradical activity, the SAHE-EPS superoxide anion scavenging activity was assessed. Briefly, riboflavin (100 μl), 200 μl EDTA, and 100 μl nitro blue tetrazolium were mixed with various quantities of SAHE-EPS (10, 50, 100, 200, and 500 μg/ml) and incubated at 25 °C for 15 min. The absorbance at a wavelength of 590 nm was determined by comparing it to a blank using an Optima spectrophotometer. The reduction in absorbance of the reaction mixture indicated an increase in the activity of scavenging superoxide anions [46]. The test was conducted in triplicate.

Percentage of superoxide anion scavenging activity = [(A_C_ - A_E_)/A_C_] × 100.

A_C_: The mean of absorbances of negative control; A_E_: The mean of absorbances of SAHE-EPS.

The interpolation method was used to determine the values by analyzing the graph of inhibition % versus sample concentration using linear regression equations (y = ax + b and R^2^).

Y = 50; a: Slope of the linear regression equation; x: IC50 value of SAHE-EPS (μg/ml) at which 50% of superoxide anion radicals are repressed; b: Intercept of linear regression equation; R^2^: Regression squared.

### SAHE-EPS hydroxyl radical scavenging activity

SAHE-EPS hydroxyl radical scavenging activity was further assessed as follows: 1 ml of a serial dilution of the SAHE-EPS (10, 50, 100, 200, and 500 μg/ml), together with either the positive control ascorbic acid or the reference compound piroxicam, was combined with 1 ml of salicylic acid, 1 ml of FeSO_4_, and 1 ml of H_2_O_2_. Subsequently, the combination was subjected to incubation for 60 min at 37° C. The absorbance was measured at a wavelength of 510 nm using an optima spectrophotometer. A decrease in absorbance implies an increase in the activity of scavenging hydroxyl free radicals [47]. The test was conducted in triplicate.

Percentage of OH scavenging activity = [(A_C_ - A_E_)/A_C_] × 100.

A_C_: The mean of absorbances of negative control; A_E_: The mean of absorbances of SAHE-EPS; It was calculated by interpolation from the graph of inhibition percentage against sample concentration using linear regression equations (y = ax + b and R^2^).

Where; Y = 50; a: Slope of the linear regression equation; x: IC50 value of SAHE-EPS(mg/ml) at which 50% of hydroxyl radicals are repressed; b: Intercept of linear regression equation; R^2^: Regression squared.

### SAHE-EPS cytotoxicity and anticancer activity

For this experiment, a volume of 200 μL of either the normal lung WI38 (ATCC: CCL-25) or the cancer cell A549 (ATCC: CCL-185) suspension, which contained 3000 cells per well, was added to each well of a 96-well plate and left to incubate for 24 h. After the initial incubation, the plate was re-incubated for 24 h in a CO_2_ incubator with specific conditions: 37 °C temperature, 5% CO_2_, and 90% relative humidity. The incubation was carried out with 100 μL of different doses of EPS in RPMI medium, excluding fetal bovine serum. After 24 h of incubation, 20 μL of MTT solution was introduced into each well. Subsequently, the plates were incubated for 3 h in a CO_2_ incubator to allow the reaction of MTT. Then, the medium was removed after plate centrifugation at a speed of 1650 rpm for 10 min. The formazan crystals, a byproduct of MTT, were dissolved again in 100 μL of DMSO, and reading was measured at a wavelength of 570 nm using an optima spectrophotometer.

### The % viability was calculated as follows: (A_T_-A_b_ /A_C_-A_b_) x 100

**A**_**T**_ = mean absorbances of cells treated with different concentrations of EPS.

**A**_**C**_ = mean absorbances of control untreated cells with culture medium only.

**A**_**b**=_ mean absorbances of cells treated with vehicle of EPS (RPMI without fetal bovine serum).

Using the percentage viability derived from the serial dilutions of each EPS concentration, the GraphPad Instat software calculated the cytotoxicity assay of the molecule and expressed it as IC50.

### Statistical analysis

The experiments were conducted in triplicate, and results were presented as the mean ± standard deviation (SD). The data was analyzed using Microsoft Excel 2010, and statistical significance was determined by applying one-way analysis of variance (ANOVA). The observed differences exhibited statistical significance at a significance level of *P* < 0.05.

## Results and discussion

### Isolation, morphological, and molecular identification of SAHE isolate

**Fig. 1:**
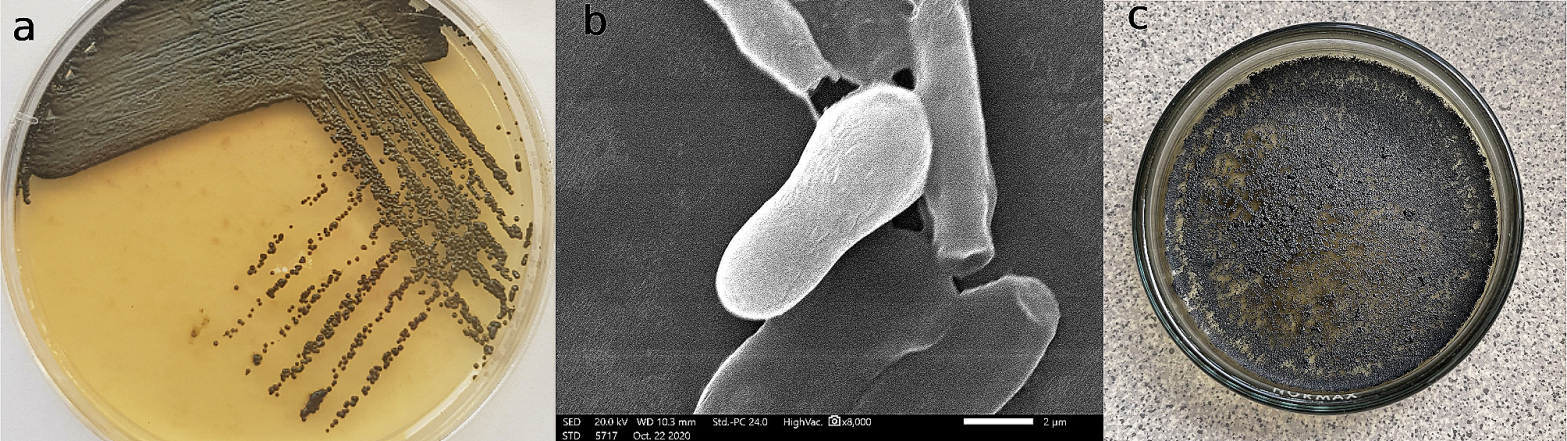
Morphology of the marine black yeast isolate SAHE cells on SDA medium (**a**), scanning electron microscopy (SEM) image of SAHE yeast cells (**b**), and dried SAHE-EPS (**c**)

The marine isolate SAHE displayed a unique characteristic of black-pigmented mucoid colonies on SDA medium supplemented with L-tyrosine (Fig. 1a) among the obtained isolates. The SEM analysis revealed that SAHE cells exhibit a columnar shape with distinct budding, characteristic of most yeasts (Fig. 1b) [32]. Therefore, this promising yeast strain was selected for EPS production (Fig. 1c) and further investigation.

**Fig. 2:**
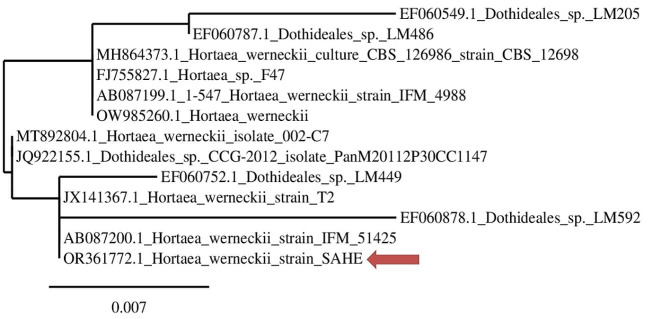
Phylogenetic tree constructed according to the ITS partial gene sequence of the black yeast strain SAHE identified as *Hortaea werneckii* by BLAST (NCBI). The tree was constructed using the website https://www.phylogeny.fr

The molecular identification of SAHE demonstrated high similarity between the isolate SAHE under investigation and *Hortaea werneckii* (identity 98.5%). The obtained sequence was deposited into the GenBank with accession number OR361772. The phylogenetic relation of *H. werneckii* SAHE and its closely related counterparts was determined and illustrated through the neighbor-joining tree in Fig. 2.

### Characterization of SAHE-EPS

#### Structural functionalization by FTIR

**Fig. 3:**
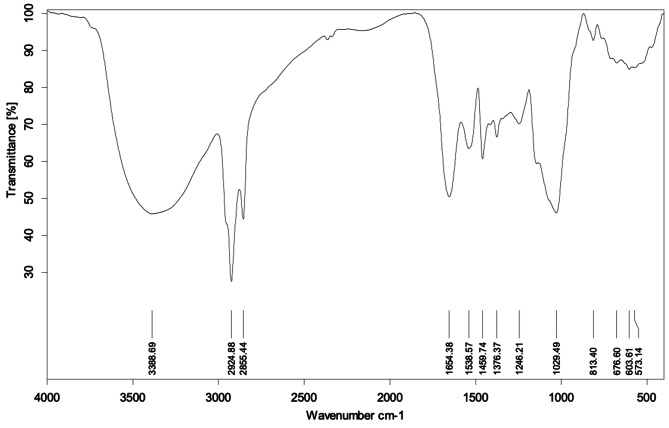
FTIR spectrum of the purified exopolysaccharide produced by *H. werneckii* SAHE

The characterization of SAHE-EPS was started by FTIR analysis to determine its main functional groups. The FTIR spectrum of *H. werneckii* SAHE EPS displayed polysaccharide-distinguishing functional groups at 4000–400 cm^− 1^ (Fig. 3).

The obtained IR spectrum displayed robust broadband at 3388 cm^− 1,^ matching the hydroxyl (O-H) group stretching vibration. The peak at 2924 cm^− 1^ corresponds to aliphatic C-H group stretching vibration, representing the polysaccharides [48]. The band at 1654 cm^− 1^ was characteristic of the C = O group involved in the H-bond [49]. The absorption area at 1400–1200 cm^− 1^ was corresponding to the flexural vibration of C-H within the polysaccharide’s structures [48] and the stretching vibration of C = O of the carboxyl (COO−) group [50]. The band at 603–813 cm^− 1^ denoted to α- and β-glycoside linkages between sugar moieties [51] with a maximum peak around 1029 cm^− 1^ apportioned to the stretching vibrations of the C-O and C-O-C groups [50]. The autograph of the IR spectrum in the current study is in accordance with other previous reports concerning the exopolysaccharides [40, 48, 52]. The FTIR analysis proposed that the produced EPS by *H. werneckii* was an acidic polysaccharide owing to the presence of a broad absorption band at 3500–3000 cm^− 1^ in addition to the appearance of bands at 1400–1200 cm^− 1^ region corresponding to the asymmetric and symmetric vibrations of carboxylate (COO−) group which is in consistence with other microbial EPSs [5, 52].

### SAHE-EPS composition by GC-MS

**Fig. 4:**
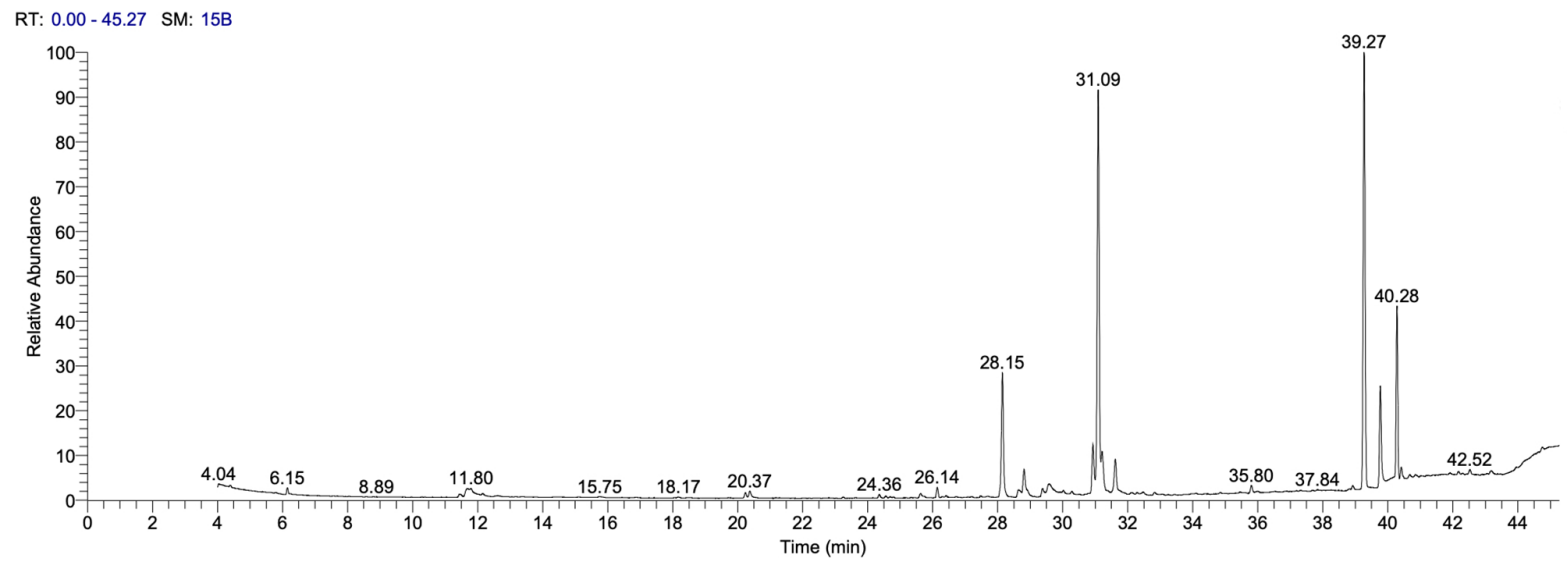
GC-MS chromatogram of the purified EPS produced by *H. werneckii* SAHE

GC-MS investigation for the derivatized EPS was performed to investigate the sugars forming the EPS. The GC-MS chromatogram (Fig. 4) revealed strong peaks in the region at 40.28 min with the identification of 3 kinds of sugar, namely maltose, cellobiose, and lactose, with an area of 8.9%, and at 39.77 and 39. 27 with the identification of one sugar, namely sucrose, with an area of 6.34% and 25.29%; respectively while mannopyranose, glucopyranose, and galactose were detected at 26.14 min with area 0.57%. These results reflect that the highest occurrence was for sucrose, representing an area of 25.29%.

The GC-MS analysis revealed SAHE-EPS’s heterogeneous nature as they are formed mainly from sucrose, maltose, cellobiose, lactose, and galactose. This result is in line with previous reports of EPS from other yeast species, namely *Rhodotorula glutinis* [53], *R. mucilaginosa* GUMS16 [54], and *Papiliotrema terrestris* PT22AV [5]. That indicates the heterogeneous nature of yeast EPS.

### Morphological and elemental investigations by SEM and EDX spectroscopy

**Fig. 5:**
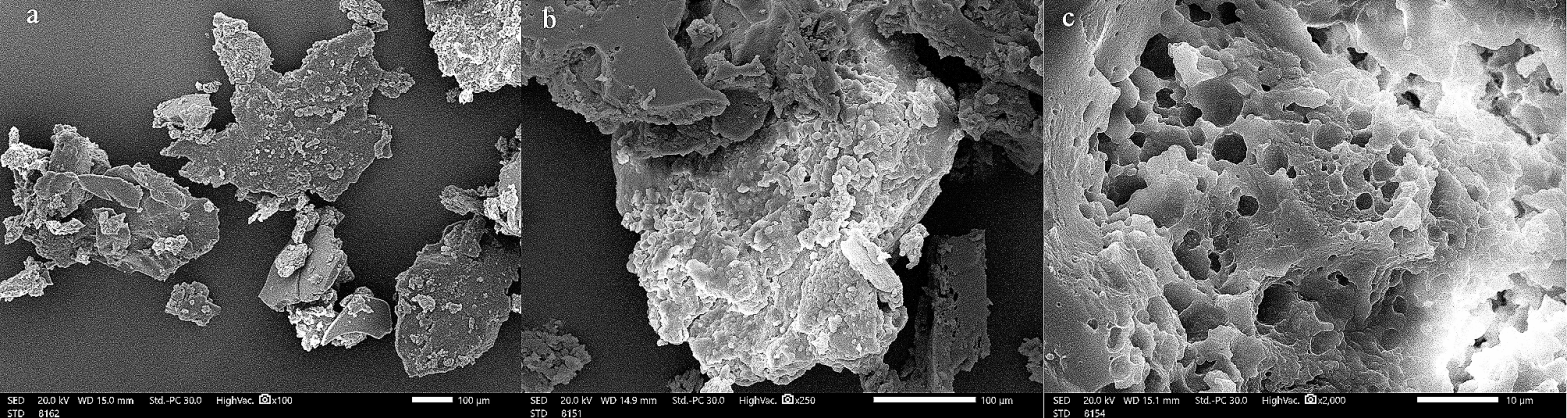
Surface morphology of *H. werneckii* SAHE EPS investigated under SEM at 100X (a), 250X (b), and 2,000X (c)

The purified SAHE-EPS SEM images revealed an irregular morphology characterized by an uneven and porous surface structure (Fig. 5). Similarly, prior studies have reported the detection of comparable morphology in different yeast EPS [55]. .

**Fig. 6:**
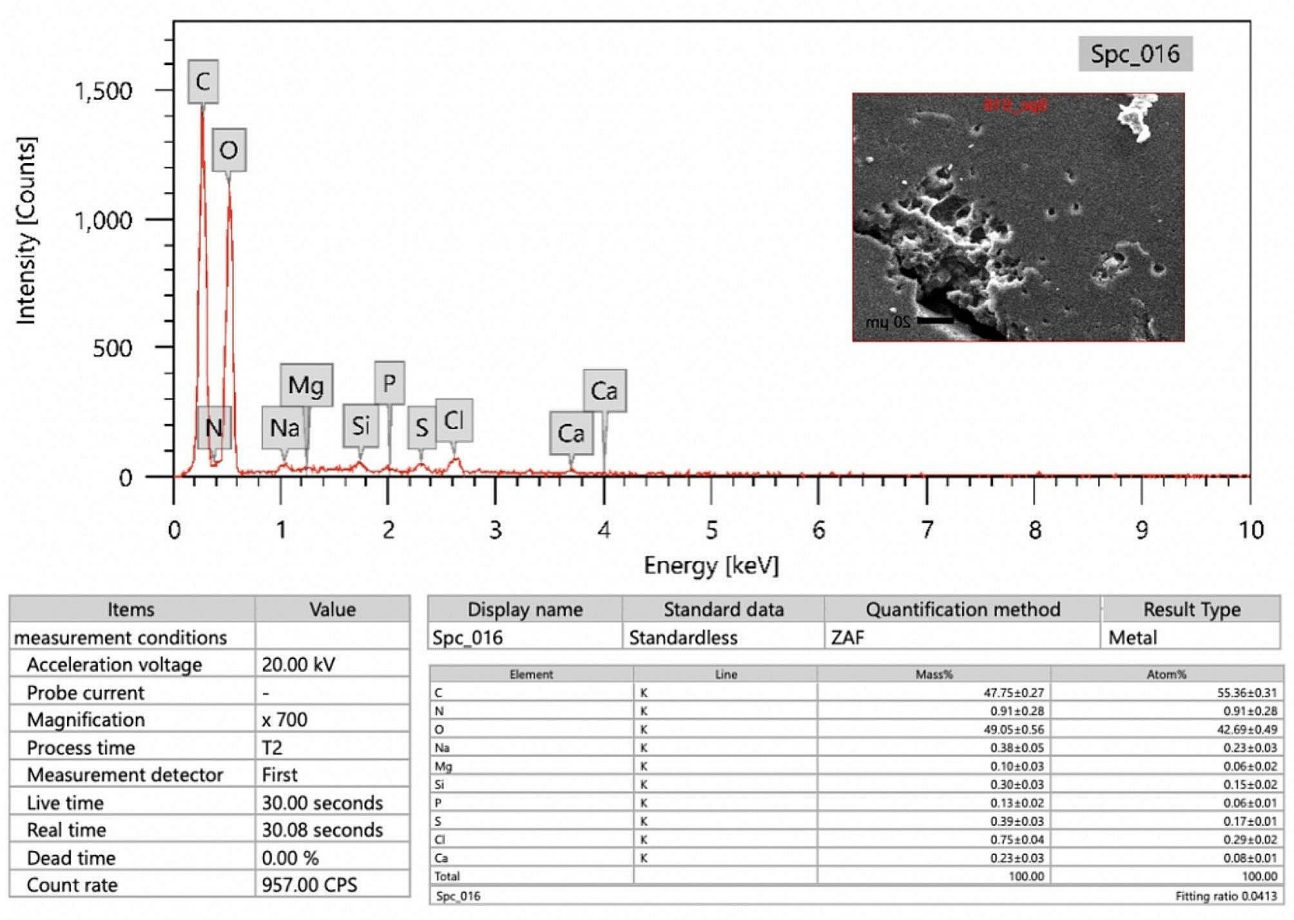
SEM-EDX analysis of the EPS produced by *H. werneckii* SAHE

At the same time, the elemental analysis of the purified EPS via EDX (Fig. 6) indicated that oxygen and carbon are the predominant constituents, comprising mass ratios (w/w %) of 55.36% and 42.69%, respectively. This observation suggests the carbohydrate nature and the efficient purification of the EPS. This confirms that the purified EPS is predominantly composed of carbohydrates. The presence of additional heteroatoms, specifically nitrogen (0.91%) and phosphorus (0.06%), also confirms the purity of the EPS, as only tiny amounts of protein and phospholipids are present in the EPS. Additional heteroatoms were also identified, including Na, Cl, Si, Mg, Ca, and S. A comparable observation was demonstrated by Zaghloul et al. [52] who observed the presence of heteroatoms such as Na, Cl, and Mg in the purified microbial EPS EDX analysis. These elements in the EPS may play a role in interacting with hydroxyl and carboxyl groups of monosaccharides [56].

### The TGA analysis

**Fig. 7:**
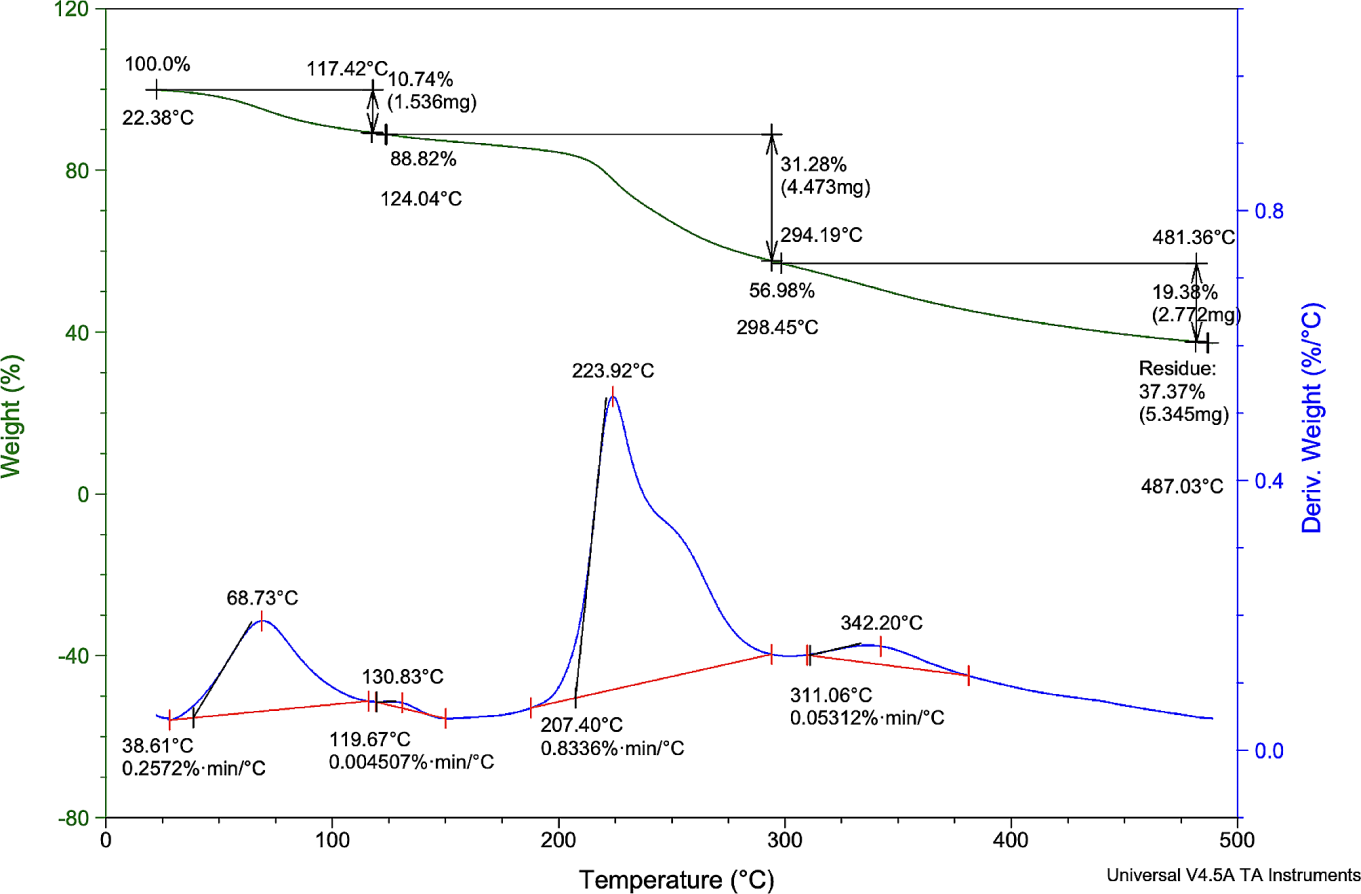
TGA analysis of the EPS produced by *H. werneckii* SAHE

The TGA analysis of SAHE-EPS was conducted with dynamism between temperature and loss of weight. As displayed in Fig. 7, the tested EPS exhibited an initial weight loss at temperatures between 124 and 294^o^C, where weight loss was about 10.74 and 31.28% at 124 and 294^o^C, respectively. Less weight loss (19%) was detected at 48^o^C. This initial weight loss may be accompanied by moisture loss and high water-bound carboxyl groups [57]. The variations between values of the degradation temperature (𝑇𝑑) of different EPS may be attributed to their diverse structure. The fact that EPS from *H. werneckii* SAHE could be subjected to a range of temperatures close to 100 ^o^C denotes its thermostability and rheological characteristics, indicating that SAHE-EPS is a promising applicant for the food industries [58]. The produced SAHE-EPS exhibited more thermostability than other microbial EPS as the initial weight loss of EPS from *Streptococcus thermophilus* CC30 was recorded to be between 50 and 99 ^o^C, and a significant loss of weight was detected at 110.84 ^o^C [59].

### Biological activity of SAHE-EPS

#### Antiradical activity

**Fig. 8:**
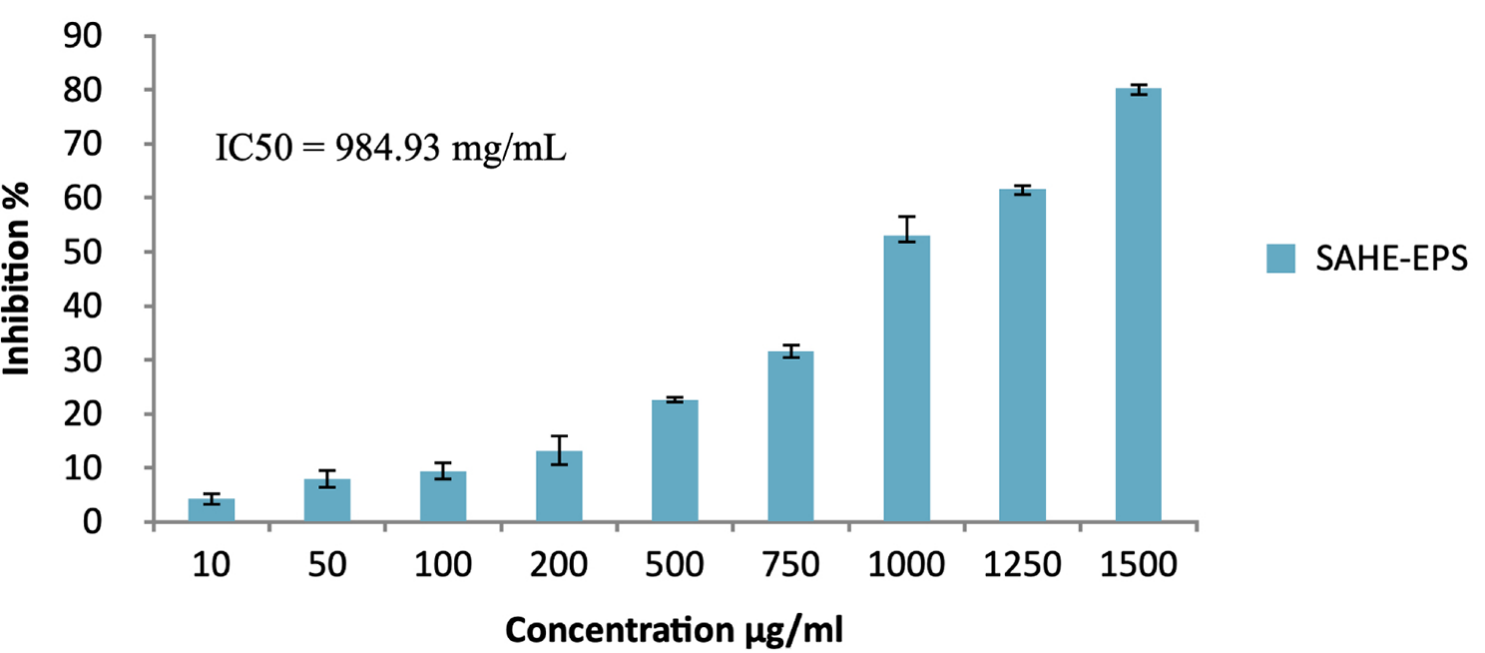
Antiradical activity of the SAHE-EPS using the DPPH radical scavenging test

**Fig. 9:**
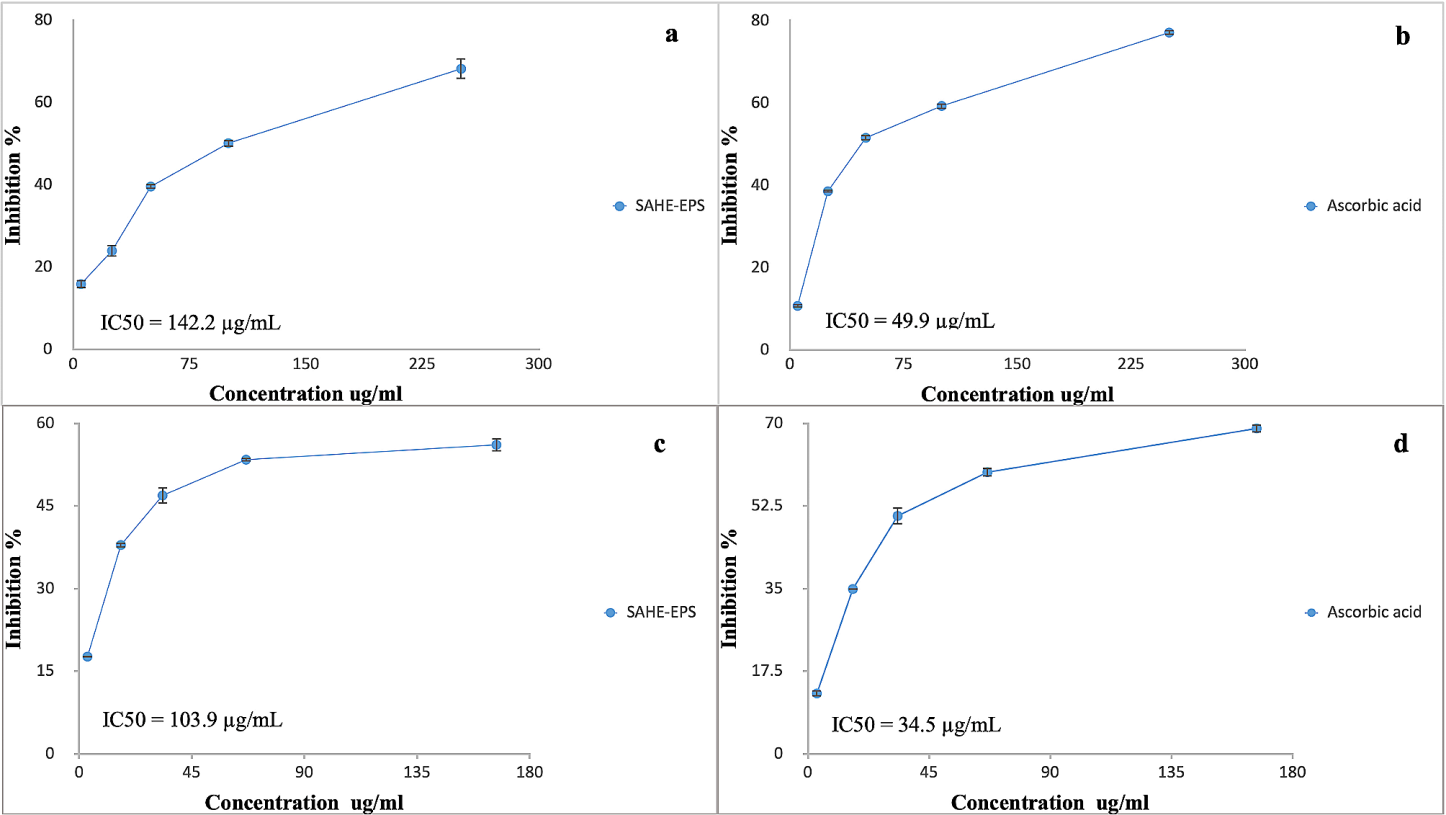
Superoxide anion scavenging activity of SAHE-EPS (**a**), Ascorbic acid (**b**), and hydroxyl radical scavenging activity of SAHE-EPS (**c**), and Ascorbic acid (**d**)

The SAHE-EPS antiradical activity was examined at various concentrations (ranging from 0.1 to 5 mg/mL) using the DPPH radical scavenging assay. Results shown in Fig. 8 showed that the DPPH scavenging activity exhibited a dose-dependent increasing manner; the highest activity (80.25%) was recorded at 1500 μg/mL, while the IC50 value was estimated to be 984.9 μg/mL. Moreover, the antiradical activity of the SAHE-EPS was further confirmed by assessing the superoxide anion scavenging and hydroxyl radical scavenging activities. SAHE-EPS showed interesting results and recorded IC50 of 142.23 and 103.9 μg/mL, respectively (Fig. 9).

The obtained results indicate that SAHE-EPS is a promising antiradical agent as higher IC50 values were reported for EPS produced by different yeast strains, namely TSPS-1 produced by *Tremella sanguinea* showed increased dose-dependent antioxidant activity of 56% at a concentration of 2.5 mg/mL, and IC50 of 1.92 mg/mL [60]. The yeast strain *Rhodotorula mucilaginosa* sp. GUMS16 exhibited a scavenging activity of 28.7 ± 2.62% at 7.5 mg/mL [54], while the yeast strain *R. babjevae* exhibited a scavenging activity of 25.2 ± 1% at a concentration of 10 mg/mL, and *R. minuta* IBRC-M 30,135 exhibited a scavenging activity of 21.8 ± 0.7% at a concentration of 10 mg/mL [9, 54].

Thus, due to its potent antiradical activity, SAHE-EPS can be recommended as a natural substitute for synthetic antiradicals in the pharmaceutical, food, and cosmetic industries. It was reported that the EPS antiradical activity primarily relies on its structural properties, encompassing factors such as the contents of monosaccharides, glycosidic linkage configuration, and molecular weight [61]. The scavenging capabilities of EPS might also be due to OH groups existing in the polysaccharides, which cause the reduction of free radicals to highly stable formulas or break the chain of free radicals through donating electrons [62].

#### Anticancer activity

**Fig. 10:**
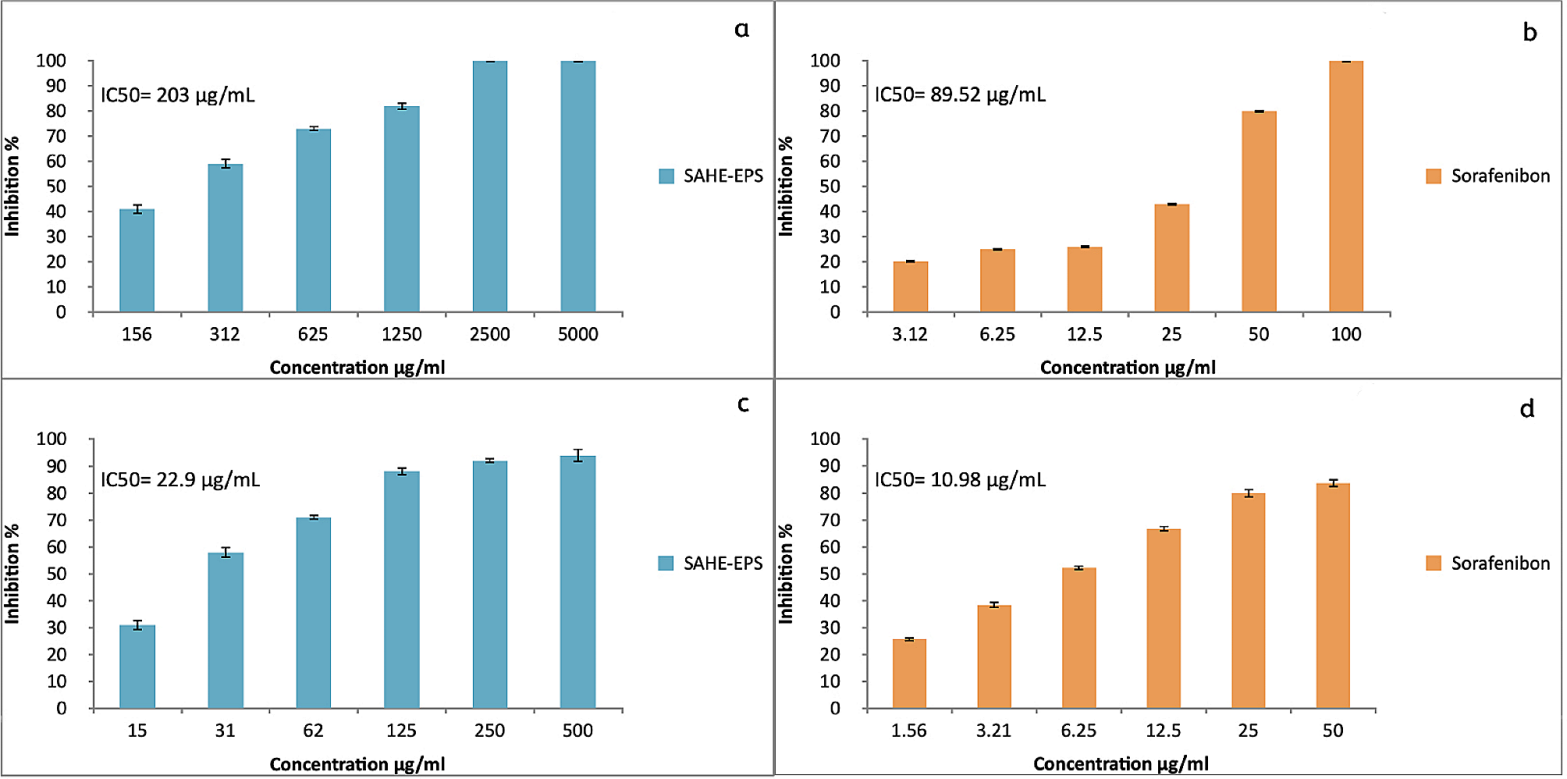
Cytotoxicity of SAHE-EPS (**a**) and Sorafenibon as a control (**b**) against normal human lung cell line (WI-38) and anticancer activity of SAHE-EPS (**c**) and Sorafenibon as a control (**d**) against human lung cancer cell line (A549)

The cytotoxicity and anticancer activity of SAHE-EPS were evaluated against normal lung cell line WI-38 and A549 human lung cancer cell line, respectively. The EPS was tested at various concentrations (ranging from 156 to 5000 μg/mL). As the EPS concentration increased, the cancer cells’ viability dropped (Fig. 10). However, different sensitivity of the tested cell lines was observed toward the tested EPS. The highest activity against A549 cancer cells of 99% was detected at 5000 μg/mL, while the IC50 was recorded to be as low as 22.9 μg/mL (Fig. S1). On the other hand, SAHE-EPS IC50 against WI-38 cells was recorded to be 203 μg/mL, indicating its safety at low concentrations and promising anticancer activities, as the Sorafenibon anticancer drug used as control recorded IC50 of 89.52 and 10.98 against WI-38 and A549 cell lines respectively.

Several studies have described EPS’s major anticancer mechanisms, including inhibiting angiogenesis, immunomodulation system preservation, apoptosis of cancer cells, and breaking the cell cycle [63].

In accordance with our results, Wu et al. [64] confirmed the effective anticancer activity of *Glehnia littoralis* polysaccharides on the A549 human lung cancer cell line in a dose- and time-dependent manner with the highest influence on growth reduction detected at 380 μg/mL. Kogan et al. [65] investigated the anticancer potentiality of polysaccharides from the yeast *Candida utilis*, showing that beta-D-glucan derivatives improved tumor necrosis factor-alpha (TNF-alpha) and exhibited the capability for treatment of 2 types of lymphosarcoma and Lewis lung carcinoma.

The chemical conformation of SAHE-EPS, which included sucrose, mannopyranose, galactose, and glucopyranose, could clarify its anti-tumor activity. For example, galactose is extensively used to support the nanocarrier in cancer therapy, which is advantageous for designated therapy of tumors. It could conjugate with platinum (II), resulting in a complex with therapeutic influence [66].

## Conclusion

The current study represents the first attempt to highlight the importance of the marine black yeast *H. werneckii* and its EPS. It achieves this through conducting a molecular taxonomic analysis of a novel black yeast strain, *H. werneckii* SAHE, using ITS1 and ITS4 gene sequencing. SAHE was used for EPS production, characterization, and assessment of its antiradical and anticancer activities. SAHE-EPS appears in the form of irregular block-shaped structures that have a rough, porous surface. It exhibited significant antiradical properties and showed promising anticancer effects on human lung cancer A549 cell line with much lower cytotoxicity against normal lung cells. In addition, SAHE-EPS showed thermostability, making it suitable for industrial applications. The examination of SAHE-EPS using FTIR, GC-MS, EDX, and SEM indicated that it primarily consists of sucrose, maltose, cellobiose, lactose, and galactose. These results indicate that *H. werneckii* SAHE could be used as a safe source of bioactive compounds for pharmacological uses. Further in vivo examination, however, is required to demonstrate the beneficial bioactivities in the food and medical sectors.

### Electronic supplementary material

Below is the link to the electronic supplementary material.

Supplementary Material 1

## Data Availability

All data are presented in the manuscript.

## References

[1] Osemwegie OO, Adetunji CO, Ayeni EA, Adejobi OI, Arise RO, Nwonuma CO, Oghenekaro AOJH. Exopolysaccharides from bacteria and fungi: current status and perspectives in Africa. 2020;6. 10.1016/j.heliyon.2020.e04205 PMC7303563 32577572

[2] Cheong K-L, Yu B, Chen J, Zhong SJF. A comprehensive review of the cardioprotective effect of marine algae polysaccharide on the gut microbiota. 2022;11:3550. 10.3390/foods11223550 PMC9689188 36429141

[3] Garza-Rodríguez ZB, Hernández-Pérez J, Santacruz A, Jacobo-Velázquez DA, Benavides JJCRiB. Prospective on the application of abiotic stresses to enhance the industrial production of exopolysaccharides from microalgae. 2022.

[4] Wu Z, Yang Z, Gan D, Fan J, Dai Z, Wang X, Hu B, Ye H, Abid M, Zeng XJB. Bioenergy: influences of carbon sources on the biomass, production and compositions of exopolysaccharides from Paecilomyces Hepiali HN1. 2014;67:260–9.

[5] Hamidi M, Okoro OV, Ianiri G, Jafari H, Rashidi K, Ghasemi S, Castoria R, Palmieri D, Delattre C, Pierre GJJoAR. Exopolysaccharide from the yeast papiliotrema terrestris PT22AV for skin wound healing. 2023;46:61–74. 10.1016/j.jare.2022.06.012 PMC10105244 35760297

[6] Casillo A, Lanzetta R, Parrilli M, Corsaro MMJM. Exopolysaccharides from marine and marine extremophilic bacteria: structures, properties, ecological roles and applications. 2018;16:69. 10.3390/md16020069 PMC5852497 29461505

[7] Banerjee A, Sarkar S, Govil T, González-Faune P, Cabrera-Barjas G, Bandopadhyay R, Salem DR, Sani RKJFiM. Extremophilic exopolysaccharides: biotechnologies and wastewater remediation. 2021;12:721365. 10.3389/fmicb.2021.721365 PMC8417407 34489911

[8] Sountharavallee VS, Kumar MS, Kathiresan K, Kavitha DJJCLM. Biosynthesis and delineate industrially prime Exopolysaccharide (MYCtPs) from marine yeast Candida Tropicalis (MYCt). 2023;11:275–85.

[9] Seveiri RM, Hamidi M, Delattre C, Rahmani B, Darzi S, Pierre G, Sedighian H, Brasselet C, Karimitabar F, Dibazar SPJMS. Characterization of the exopolysaccharides from Rhodotorula minuta IBRC-M 30135 and evaluation of their emulsifying, antioxidant and antiproliferative activities. 2019;23:381–9.

[10] Yadav KL, Rahi DK, Soni SKJAb. Biotechnology: an indigenous hyperproductive species of Aureobasidium Pullulans RYLF-10: influence of fermentation conditions on exopolysaccharide (EPS) production. 2014;172:1898–908. 10.1007/s12010-013-0630-3 24293276

[11] Gientka I, Bzducha-Wróbel A, Stasiak-Różańska L, Bednarska AA, Błażejak SJEJoB. The exopolysaccharides biosynthesis by Candida yeast depends on carbon sources. 2016;22:31–7.

[12] Yildiran H, BAŞYİĞİT KILIÇ G, Karahan ÇAKMAKÇI AGJFS. Technology: characterization and comparison of yeasts from different sources for some probiotic properties and exopolysaccharide production. 2019;39:646–53.

[13] Rahbar Saadat Y, Yari Khosroushahi A, Pourghassem Gargari BJFM. Yeast exopolysaccharides and their physiological functions. 2021;66:171–82. 10.1007/s12223-021-00856-2 33604744

[14] Qi M, Zheng C, Wu W, Yu G, Wang PJMD. Exopolysaccharides from marine microbes: source, structure and application. 2022;20:512. 10.3390/md20080512 PMC9409974 36005515

[15] Sakkaa SE, Zaghloul EH, Ghanem KMJP. Proteins A: psychobiotic potential of gamma-aminobutyric acid–producing marine enterococcus faecium SH9 from marine shrimp. 2022;14:934–46. 10.1007/s12602-022-09963-z PMC9474364 35750975

[16] Concórdio-Reis P, Alves VD, Moppert X, Guézennec J, Freitas F, Reis MAJMd. Characterization and biotechnological potential of extracellular polysaccharides synthesized by Alteromonas strains isolated from French polynesia marine environments. 2021;19:522. 10.3390/md19090522 PMC8470090 34564184

[17] Concórdio-Reis P, Serafim B, Pereira JR, Moppert X, Guézennec J, Reis MA, Freitas FJET. Innovation: exopolysaccharide production by the marine bacterium Alteromonas macleodii Mo169 using fruit pulp waste as the sole carbon source. 2023;30:103090.

[18] Roca C, Lehmann M, Torres CA, Baptista S, Gaudêncio SP, Freitas F, Reis MAJNb. Exopolysaccharide production by a marine Pseudoalteromonas sp. strain isolated from Madeira Archipelago ocean sediments. 2016;33:460–6. 10.1016/j.nbt.2016.02.005 26923806

[19] Hamed SB, Belhadri M (2009). Rheological properties of biopolymers drilling fluids. J Petrol Sci Eng.

[20] Farwick M, Lersch P, Schmitz G, Müllner S, Wattenberg A, Protagen A. Skin-omics: use of genomics, proteomics and lipidomics to assess effects of low molecular weight scleroglucan. Cos Sci Technol E Indus 2009:100–5.

[21] Bar-Zeev E, Rahav EJF. Microbial metabolism of transparent exopolymer particles during the summer months along a eutrophic estuary system. 2015;6:403. 10.3389/fmicb.2015.00403 PMC4436900 26042092

[22] Decho AW, Gutierrez TJFim. Microbial extracellular polymeric substances (EPSs) in ocean systems. 2017;8:922. 10.3389/fmicb.2017.00922 PMC5445292 28603518

[23] Gunde-Cimerman N, Zalar PJFT. Biotechnology: extremely halotolerant and halophilic fungi inhabit brine in solar salterns around the globe. 2014;52:170–9.

[24] Campana R, Fanelli F, Sisti MJFB. Role of melanin in the black yeast fungi Aureobasidium pullulans and Zalaria obscura in promoting tolerance to environmental stresses and to antimicrobial compounds. 2022;126:817–25. 10.1016/j.funbio.2022.11.002 36517149

[25] Chung D, Kim H, Choi HSJJM. Fungi in salterns. 2019;57:717–724. 10.1007/s12275-019-9195-3 31452042

[26] Elsayed A, Mowafy AM, Soliman HM, Gebreil A. Magdy NIJEjob. Sciences A: characterization of new strains of Hortaea Werneckii isolated from salt marshes of Egypt. 2016;3:350–6.

[27] Brauers G, Ebel R, Edrada R, Wray V, Berg A, Gräfe U, Proksch PJJonp. Hortein, a new natural product from the fungus Hortaea w Erneckii associated with the Sponge Aplysina a erophoba. 2001;64:651–2. 10.1021/np000542u 11374967

[28] Plemenitaš A, Vaupotič T, Lenassi M, Kogej T, Gunde-Cimerman NJSM. Adaptation of extremely halotolerant black yeast Hortaea Werneckii to increased osmolarity: a molecular perspective at a glance. 2008;61:67–75. 10.3114/sim.2008.61.06 PMC2610308 19287528

[29] Mok W, Castelo F, Barreto Da Silva MJIJoFS. Technology: occurrence of Exophiala Werneckii on salted freshwater fish Osteoglossum bicirrhosum. 1981;16:505–12.

[30] Kogej T, Stein M, Volkmann M, Gorbushina AA, Galinski EA, Gunde-Cimerman NJM. Osmotic adaptation of the halophilic fungus Hortaea Werneckii: role of osmolytes and melanization. 2007;153:4261–73. 10.1099/mic.0.2007/010751-0 18048939

[31] Badali H, Al-Hatmi AM, Fakhim H, Moghaddasi A, Khodavaisy S, Vaezi A, Ahangarkani F, de Hoog GS, Meis JFJIjoaa. In vitro activity of nine antifungal agents against a global collection of Hortaea werneckii isolates, the agent of tinea nigra. 2019;54:95–98. 10.1016/j.ijantimicag.2019.05.006 31071468

[32] Elsayis A, Hassan SW, Ghanem KM, Khairy, HJBm. Optimization of melanin pigment production from the halotolerant black yeast Hortaea Werneckii AS1 isolated from solar salter in Alexandria. 2022;22:92. 10.1186/s12866-022-02505-1 PMC8991569 35395716

[33] White TJ, Bruns T, Lee S, Taylor JJPpagtm. Applications: amplification and direct sequencing of fungal ribosomal RNA genes for phylogenetics. 1990;18:315–22.

[34] Rusinova-Videva S, Ognyanov M, Georgiev Y, Kambourova M, Adamov A, Krasteva VJAS. Production and Chemical characterization of Exopolysaccharides by Antarctic yeasts Vishniacozyma victoriae and Tremellomycetes Sp. 2022;12:1805.

[35] Padmanaban S, Balaji N, Muthukumaran C, Tamilarasan KJB. Statistical optimization of process parameters for exopolysaccharide production by Aureobasidium pullulans using sweet potato based medium. 2015;5:1067–73. 10.1007/s13205-015-0308-3 PMC4624145 28324414

[36] Zaghloul EH, Ibrahim MIJFM. Production and characterization of exopolysaccharide from newly isolated marine probiotic lactiplantibacillus plantarum EI6 with in vitro wound healing activity. 2022;13:903363. 10.3389/fmicb.2022.903363 PMC9164304 35668753

[37] Amer MS, Zaghloul EH, Ibrahim MIJTEJAR. Characterization of exopolysaccharide produced from marine-derived aspergillus terreus SEI with prominent biological activities. 2020;46:363–9.

[38] Zhai Z, Chen A, Zhou H, Zhang D, Du X, Liu Q, Wu X, Chen L, Hu F, Liu YJIJBM. Structural characterization and functional activity of an exopolysaccharide secreted by Rhodopseudomonas palustris GJ-22. 2021;167:160–8. 10.1016/j.ijbiomac.2020.11.165 33249155

[39] Yang X, Ren Y, Zhang L, Wang Z, Li LJL. Structural characteristics and antioxidant properties of exopolysaccharides isolated from soybean protein gel induced by lactic acid bacteria. 2021;150:111811.

[40] Zaghloul H EHA, Ibrahim HJEJoAB. Fisheries: comparative study on antimicrobial activity of commercial and extracted chitin and chitosan from Marsupenaeus japonicus shells. 2019;23:291–302.

[41] Mao W, Zang X, Li Y, Zhang HJJA. Sulfated polysaccharides from marine green algae Ulva conglobata and their anticoagulant activity. 2006;18:9–14.

[42] Ruiz-Matute AI, Hernández-Hernández O, Rodríguez-Sánchez S, Sanz ML, Martínez-Castro IJJoCB. Derivatization of carbohydrates for GC and GC–MS analyses. 2011;879:1226–40. 10.1016/j.jchromb.2010.11.013 21186143

[43] Ozturk Urek R, Ilgin SJAM. Production and partial characterization of the exopolysaccharide from Pleurotus Sajor Caju. 2019;69:1201–10.

[44] Ibrahim HA, Abdel-Latif HH, Zaghloul EHJTEJoAR. Phytochemical composition of Avicennia marina leaf extract, its antioxidant, antimicrobial potentials and inhibitory properties on Pseudomonas fluorescens biofilm. 2022;48:29–35.

[45] Braca A, De Tommasi N, Di Bari L, Pizza C, Politi M, Morelli IJJonp. Antioxidant principles from bauhinia t arapotensis. 2001;64:892–895. 10.1021/np0100845 11473417

[46] Ravishankara M, Shrivastava N, Padh H, Rajani M (2002). Evaluation of antioxidant properties of root bark of Hemidesmus Indicus R. Br.(Anantmul). Phytomedicine.

[47] Smirnoff N, Cumbes QJ (1989). Hydroxyl radical scavenging activity of compatible solutes. Phytochemistry.

[48] Choudhuri I, Khanra K, Pariya P, Maity GN, Mondal S, Pati BR, Bhattacharyya NJCM. Structural characterization of an exopolysaccharide isolated from Enterococcus faecalis, and study on its antioxidant activity, and cytotoxicity against HeLa cells. 2020;77:3125–35. 10.1007/s00284-020-02130-z 32725340

[49] Zhou K, Zeng Y, Yang M, Chen S, He L, Ao X, Zou L, Liu SJCP. Production, purification and structural study of an exopolysaccharide from Lactobacillus plantarum BC-25. 2016;144:205–14. 10.1016/j.carbpol.2016.02.067 27083810

[50] Sirin S, Aslim BJSR. Characterization of lactic acid bacteria derived exopolysaccharides for use as a defined neuroprotective agent against amyloid beta1–42-induced apoptosis in SH-SY5Y cells. 2020;10:8124. 10.1038/s41598-020-65147-1 PMC7229009 32415207

[51] Coimbra MA, Barros A, Rutledge DN, Delgadillo IJCR. FTIR spectroscopy as a tool for the analysis of olive pulp cell-wall polysaccharide extracts. 1999;317:145–54.

[52] Zaghloul EH, Ibrahim MI, Zaghloul HAJB. Antibacterial activity of exopolysaccharide produced by bee gut-resident Enterococcus sp. BE11 against marine fish pathogens. 2023;23:231. 10.1186/s12866-023-02977-9 PMC10463787 37612642

[53] Gientka I, Błażejak S, Stasiak-Różańska L, Chlebowska-Śmigiel AJASPTA. Exopolysaccharides from yeast: insight into optimal conditions for biosynthesis, chemical composition and functional properties? Review. 2015;14:283–92. 10.17306/J.AFS.2015.4.29 28068035

[54] Hamidi M, Gholipour AR, Delattre C, Sesdighi F, Seveiri RM, Pasdaran A, Kheirandish S, Pierre G, Kozani PS, Kozani PSJIjobm. Production, characterization and biological activities of exopolysaccharides from a new cold-adapted yeast: Rhodotorula mucilaginosa sp. GUMS16. 2020;151:268–77. 10.1016/j.ijbiomac.2020.02.206 32087227

[55] Kharat PP, RamsaranYadav S, Ragavan ML, Das NJRJoP. Technology: isolation and characterization of exopolysaccharides from yeast isolates. 2018;11:537–42.

[56] Prajapati D, Bhatt A, Gupte AJJoAB. Biotechnology: purification and physicochemical characterization of exopolysaccharide produced by a novel brown-rot fungus Fomitopsis Meliae AGDP-2. 2022;10:158–66.

[57] Wang J, Zhao X, Tian Z, Yang Y, Yang ZJC. Characterization of an exopolysaccharide produced by Lactobacillus plantarum YW11 isolated from Tibet Kefir. 2015;125:16–25. 10.1016/j.carbpol.2015.03.003 25857955

[58] Sajna KV, Sukumaran RK, Gottumukkala LD, Jayamurthy H, Dhar KS, Pandey AJIJBM (2013). Studies on structural and physical characteristics of a novel exopolysaccharide from Pseudozyma Sp. NII.

[59] Kanamarlapudi SLRK, Muddada SJBri. Characterization of exopolysaccharide produced by Streptococcus thermophilus CC30. 2017;2017. 10.1155/2017/4201809 PMC5549498 28815181

[60] Liu Y, Chen S, Zhang J, Gao M, Li LJM. Purification of Polysaccharide produced by the haploid yeast strain of Tremella sanguinea and its antioxidant and prebiotic activities. 2023;28:5391. 10.3390/molecules28145391 PMC10386508 37513263

[61] Zheng J-Q, Wang J-Z, Shi C-W, Mao D-B, He P-X, Xu C-PJPB. Characterization and antioxidant activity for exopolysaccharide from submerged culture of Boletus aereus. 2014;49:1047–53.

[62] Aruoma, OIJJotAocs. Free radicals, oxidative stress, and antioxidants in human health and disease. 1998;75:199–212. 10.1007/s11746-998-0032-9 PMC7101596 32287334

[63] Guo R, Chen M, Ding Y, Yang P, Wang M, Zhang H, He Y, Ma HJFiN. Polysaccharides as potential anti-tumor biomacromolecules—a review. 2022;9:838179. 10.3389/fnut.2022.838179 PMC8919066 35295918

[64] Wu J, Gao W, Song Z, Xiong Q, Xu Y, Han Y, Yuan J, Zhang R, Cheng Y, Fang JJIJoBM. Anticancer activity of polysaccharide from Glehnia littoralis on human lung cancer cell line A549. 2018;106:464–72. 10.1016/j.ijbiomac.2017.08.033 28797819

[65] Kogani G, Pajtinka M, Babincova M, Miadokova E, Rauko P, Slamenova D, Korolenko TJN. Yeast cell wall polysaccharides as antioxidants and antimutagens: can they fight cancer? Minireview. 2008;55:387. 18665748

[66] Wu M, Li H, Liu R, Gao X, Zhang M, Liu P, Fu Z, Yang J, Zhang-Negrerie D, Gao QJEjomc. Galactose conjugated platinum (II) complex targeting the Warburg effect for treatment of non-small cell lung cancer and colon cancer. 2016;110:32–42. 10.1016/j.ejmech.2016.01.016 26807543

