# Supplementary material for: Production and characterization of novel marine black yeast’s exopolysaccharide with potential antiradical and anticancer prospects
Source: Microb Cell Fact. 2024 Feb 22;23:60. doi: 10.1186/s12934-024-02332-1 (PMC10882794; doi:10.1186/s12934-024-02332-1)
Supplement: Supplementary file 1 — Supplementary Material 1 [file 12934_2024_2332_MOESM1_ESM.pdf]

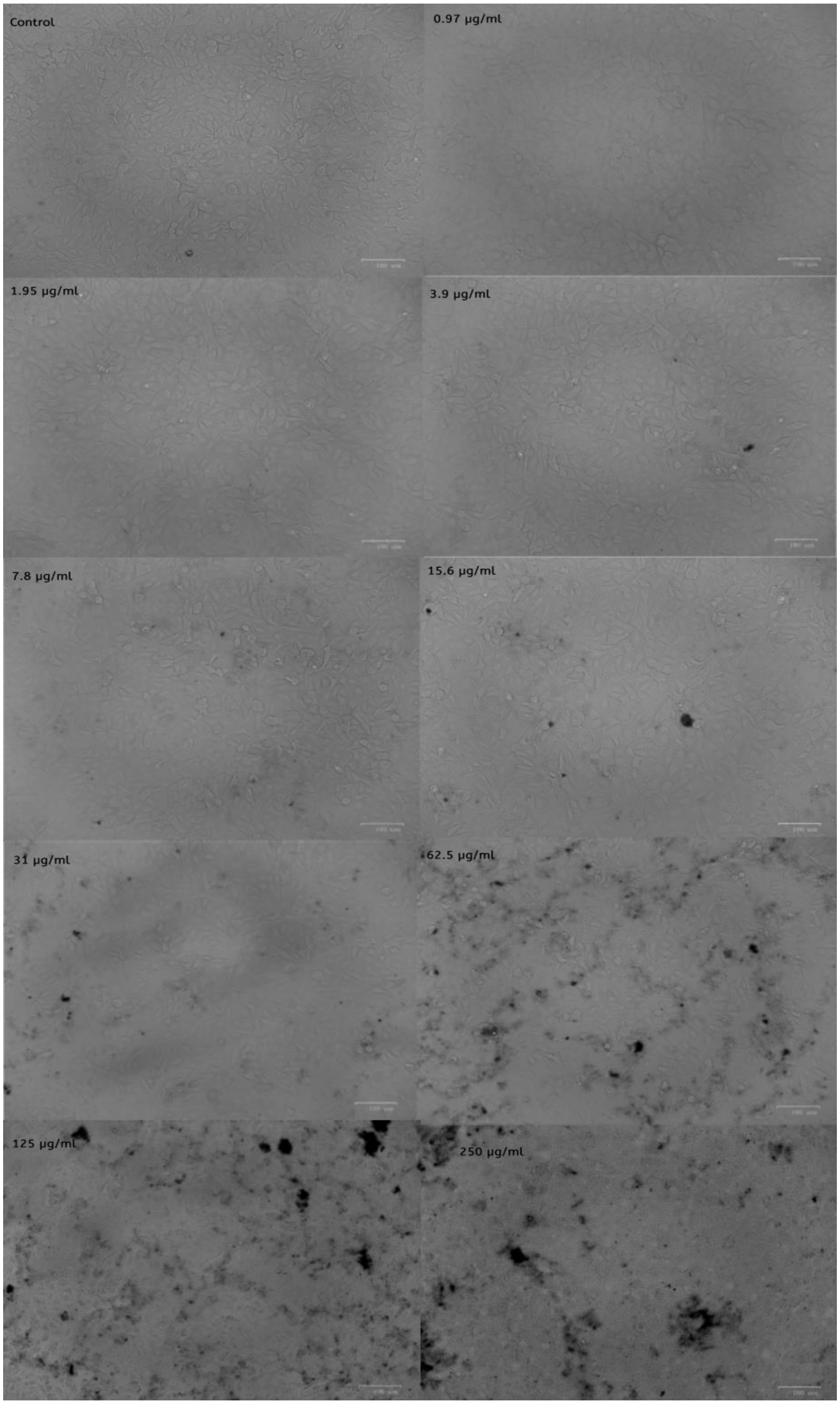

Fig. S1 Anticancer activity of SAHE-EPS against human lung cancer cell lines (A549) with different concentrations.
